# Platelet Endothelial Aggregation Receptor 1 Polymorphism Is Associated With Functional Outcome in Small-Artery Occlusion Stroke Patients Treated With Aspirin

**DOI:** 10.3389/fcvm.2021.664012

**Published:** 2021-09-01

**Authors:** Zhizhang Li, Huayu Jiang, Ying Ding, Dong Zhang, Xiaoguang Zhang, Jie Xue, Ruinan Ma, Liang Hu, Yunhua Yue

**Affiliations:** Department of Neurology, Yangpu Hospital Tongji University School of Medicine, Shanghai, China

**Keywords:** stroke, small-artery occlusion, gene polymorphism, aspirin assistance, PEAR1

## Abstract

**Background:** The role of genetic polymorphisms is important in defining the patient's prognosis and outcomes in coronary artery disease. The present study aimed to explore the association between platelet endothelial aggregation receptor 1 (PEAR1) rs12041331 polymorphism and the outcomes in patients with acute ischemic stroke treated with aspirin or dual antiplatelet therapy (DAPT) with clopidogrel.

**Methods:** A total of 868 ischemic stroke patients admitted to our hospital from January 1, 2016 to December 30, 2018 were retrospectively studied. The Trial of Org 10172 in Acute Stroke Treatment (TOAST) classification defined stroke subtypes. These patients were treated with aspirin alone or DAPT. The genotype distribution of PEAR1 rs12041331 single-nucleotide polymorphism (AA, AC, and CC) between different TOAST subtypes and treatment groups was assessed, and the clinical impact of genetic variants on functional outcomes defined by the National Institutes of Health Stroke Scale, modified Rankin Scale, and Barthel Index was analyzed using univariate and multivariate logistic regression models.

**Results:** Among the 868 stroke patients, the PEAR1 AA genotype was 16%, GA was 47%, and GG was 36%. Forty-four percent had aspirin alone, and 56% had DAPT. Overall, the distribution of PEAR single-nucleotide polymorphism was not significant among the two treatment groups or subtypes of TOAST. In contrast, in patients treated with aspirin alone, PEAR1 AA tended to be higher in the small-artery occlusion (SAO) subtype when compared with the no-lacunar subtype, including cardioembolism and large-artery atherosclerosis. PEAR1 AA genotype was significantly associated with favorable functional outcomes at day 7 and discharge only in SAO patients treated with aspirin alone compared with the GG genotype. Multivariate regression models further suggested that AA genotype was independently associated with favorable outcomes in this group after being adjusted for three common stroke risk factors such as age, hypertension history, and C-reactive protein level [odds ratio (OR) 0.23, 95% confidence interval (CI), 0.07–0.64, *P* = 0.02 for 7-day National Institutes of Health Stroke Scale; OR 0.2, 95% CI, 0.06–0.66, *P* = 0.03 for 7-day modified Rankin Scale, and OR 0.25, 95% CI, 0.08–0.72, *P* = 0.03 for 7-day Barthel Index, respectively].

**Conclusion:** The impact of PEAR1 rs12041331 polymorphism on aspirin depends on the TOAST subtype. PEAR1 AA carrier with SAO stroke is most sensitive to aspirin therapy. PEAR1 AA is an independent factor for the short-term functional outcomes in SAO patients treated with aspirin alone.

**Clinical Registration Number:** 1800019911.

## Introduction

In recent years, the number of patients with acute coronary syndrome in China has increased year by year, and according to China Stroke Statistics 2019, the death rate for cerebrovascular diseases in China was 149.49 per 100,000, accounting for 1.57 million deaths in 2018. Stroke was the leading cause of all-age disability-adjusted life years in 2017 (1). The majority (87%) of stroke patients were ischemic strokes (2). Stroke is a heterogeneous syndrome, and a wide variety of factors influence stroke prognosis, including age, stroke severity, stroke mechanism, infarct location, comorbid conditions, and interventions, such as drug treatment. Recent research also suggests that the role of genetic polymorphisms is important in defining the patient's prognosis and outcomes with coronary artery disease, including stroke (3, 4), whereas whether such influence is an independent risk factor for stroke or stroke recovery is an open debate (5, 6). Knowledge of the important factors that affect prognosis is necessary for the clinician to make a reasonable prediction for individual patients, to provide a rational approach to personalized treatment.

Antiplatelet aggregation is the main clinical treatment for ischemic stroke, and aspirin is a classical antiplatelet drug. In clinical practice, some stroke patients do not achieve the expected effect of inhibiting platelet aggregation despite taking aspirin regularly according to professional guidelines or experience, and aspirin resistance often greatly reduces the treatment efficacy and increases the risk of ischemic cardiovascular adverse events (7). Aspirin resistance is multifactorial in origin and involves diverse environmental and genetic factors, including single-nucleotide polymorphisms (SNPs) (8). Platelet endothelial aggregation receptor 1 (PEAR-1) is a platelet transmembrane protein expressed mainly in platelets and endothelial cells, promoting platelet adhesion and aggregation, and has a role in thrombosis and maintenance of platelet aggregation homeostasis. Platelet endothelial aggregation receptor 1 (PEAR1) rs12041331 has been recently associated with aspirin-alone or dual antiplatelet therapy (DAPT) response, although the association between rs12041331 and stroke outcome is not fully determined (9–12). This study aims to investigate the effect of PEAR-1 rs12041331 polymorphism in stroke patients based on etiology classified by the Trial of Org 10172 in Acute Stroke Treatment (TOAST) and the association with the short-term functional outcomes after aspirin alone and DAPT.

## Materials and Methods

### Study Population and Outcome Measures

This retrospective study comprises 868 acute ischemic stroke patients admitted to our hospital from January 1, 2016 to December 30, 2018 and received either aspirin alone (81 mg daily) or DAPT (aspirin 81 mg and clopidogrel 75 mg daily). The mean days of hospitalization were 25.6 (±37.1) days. Etiological stroke subtypes were classified according to the TOAST system (13), including large-artery atherosclerosis (LAA), small-artery occlusion (SAO), and cardioembolism (CE). The primary endpoints were functional outcomes assessed with three popular measures: the National Institute of Health Stroke Scale (NIHSS), the modified Rankin Scale (mRS), and the Barthel Index (BI) (14) when patients were admitted to the hospital, day 7 after first treatments, and at discharge. Stroke severity was defined by using the NIHSS scale system. Patients with an NIHSS score ≥1 were defined as having a poor outcome, otherwise a good outcome (15). mRS was used to evaluate the disability, with six grades from 0 to 5. Patients with an mRS score ≤ 1 were defined as having a good outcome, and patients with an mRS score >1 were defined as having a poor outcome (16). The BI is a standard and well-validated scale that measures independence in personal 10 basic activities of daily living, scoring 0–100 with five-point increments. BI ≥95 was defined as a good outcome (16). A total of 11 conventional risk factors, including age, sex, body mass index, smoking history, drinking history, history of diabetes, hypertension, atrial fibrillation, coronary artery disease, systolic blood pressure, and lipid levels, such as triglyceride, cholesterol, high-density lipoprotein (HDL), and low-density lipoprotein (LDL), were collected at admission. The formula for body mass index is weight in kilograms divided by height in meters squared. All participants gave written informed consent. The ethics committee of Yangpu Hospital, Tongji University School of Medicine approved the study.

### Genotype Testing

The gene frequencies of patients were analyzed according to the SNP method (17), and genome DNA was extracted from 3 ml of fasted peripheral blood from patients fasted for 12 h and then collected in ethylenediaminetetraacetic acid anticoagulation tubes. PEAR1 SNP Database rs12041331 (G>A) was detected by hybridization-based *in situ* sequencing (TL988A, Xi'an TianLong). The whole process was conducted according to the manufacturer's instructions. Briefly, the white blood cells (WBCs) were enriched by centrifugation using 200 μl of whole blood in 1.5 ml of ammonium chloride, at 700 × g for 5 min at room temperature. The precipitate was resuspended in 1 ml of ammonium chloride, and the mixture was centrifuged again at 700 × g for 5 min at room temperature. The nucleic acid extraction kit (Sino-Era Jiyin Tech Co., Ltd., Beijing, China) was used to extract the DNA, as per the manufacturer's instructions. After 15 min of stasis, universal sequencing kits (Sino-Era Jiyin Tech Co., Ltd.) were added to 2 μl of the sample, and PEAR1 rs12041331 polymorphisms were then identified by hybridization-based *in situ* sequencing using fluorescent probes (18, 19) (Tianlong Science and Technology Co., Ltd., Xi'an, China). The target sequence (5′-3′) is GTCTCACTTCC (G/A)TCACCCTTACTC.

### Laboratory Measurements

Blood sampling was performed within 24–48 h of the stroke event or at admission. Venous blood samples were drawn between 8:30 and 10:30 am after an overnight fast. C-reactive protein (CRP), total triglyceride, cholesterol, HDL, and LDL were analyzed with standard assays on a D × C 700 AU analyzer (Beckman Coulter, Brea, CA, USA). Counts of WBC, neutrophil, and lymphocyte were analyzed on an Advia 2120 analyzer (Siemens Diagnostics, Malvern, PA, USA). Interleukin-6 (IL-6) was determined with chemiluminescent microparticle immunoassay on an MLX Microtiter luminometer (Dynex Technologies, Chantilly, VA, USA). As for platelet aggregation inhibition rate, fasting peripheral blood was drawn from patients at least 7 days after the administration of the first treatment, and then a thromboelastography platelet mapping (TEG-PM) analyzer (Haemoscope, Model 5000) was used to detect platelet function. The analyzer measured the percent inhibition of the platelet activated by adenosine diphosphate (ADP) and arachidonic acid. This parameter was calculated by comparing the maximum amplitude (MA), representing the viscoelastic strength of the thrombus, created through three TEG channels as follows: (1) The MA reflects thrombin activated platelets (MAThrombin) measured by a kaolin/Ca^2+^-activated citrated blood sample; (2) a reptilase and activator F-activated heparinized blood sample, representing a fibrin-only contribution to the clot strength (MAFibrin); and (3) the heparinized blood mixed with 2-mM ADP (MAADP) or 1-mM AA (MAAA) combined with activator F as platelet agonists reflect platelet responsiveness to ADP and arachidonic acid. The equation 100 - ((MAADP or MAAA) - MAFibrin)/(MAThrombin - MAFibrin)) × 100 was used to calculate the percentage of platelet inhibition in response to ADP and arachidonic acid (20). Arachidonic acid or ADP-induced platelet inhibition rate ≤ 30% was considered aspirin or clopidogrel resistant (21, 22).

### Statistical Analysis

Demographic data were analyzed using descriptive methods, with the mean ± standard deviation or the median. Comparison of differences between or among different groups, including PEAR1 SNPs, TOAST subtypes, and drug treatments, was made using one-way analysis of variance for continuous variables and chi-square tests for categorical variables (with continuity correction). Reported significant *P*-values were further adjusted using false discovery rate (FDR) correction for multiple testing. Allele frequencies were estimated by gene counting, and Hardy–Weinberg equilibrium was tested using the chi-square test. Univariate and multivariate logistic regression models were used for outcome association analysis using outcomes defined by dichotomized NIHSS, mRS, and BI scoring systems. Missing values for lab measurements such as levels of CRP, IL-6, total triglyceride, cholesterol, HDL, LDL, counts of WBC, neutrophil, and lymphocyte were replaced by the median value to have the possibility of using all available clinical information in the regression model (23). TOAST subtypes were further divided into non-lacunar (CE or LAA) and lacunar (SAO) (24) in logistic regression models. The association among the proportions of TOAST subtypes, treatment groups, and PEAR1 SNP were assessed using the Cochran–Mantel–Haenszel test. Variables selection was made using best subset regression, and a bootstrap-validated bias-correction approach was further used to evaluate the regression models (25, 26). Odds ratios (ORs) and 95% confidence intervals (95% CIs) were calculated for these parameters. An FDR method was also used to correct *P*-values after multiple comparisons in regression models (27). Logarithmically transformed CRP and IL-6 levels were used in the logistic regression model. Data were analyzed in the R 4.1 environment. Statistical analyses were performed in a two-tailed fashion. A *P* < 0.05 or FDR < 0.05 was considered to be statistically significant.

### Data Availability

The processed data that support the findings of this study are available on request from the corresponding author YY.

## Results

### Characteristics of Patients by Platelet Endothelial Aggregation Receptor 1 Single-Nucleotide Polymorphisms

This retrospective study included a total of 868 subjects with a mean age of 71 years (±12 years). Table 1 describes the baseline clinical characteristics of the overall population classified by PEAR1 rs12041331 polymorphism, including homozygous AA (16%), heterozygous GA (48%), and wild-type GG (36%). The genotype of the rs12041331 SNP followed the Hardy–Weinberg equilibrium in our study (χ^2^ = 0.005, *P* = 0.94). Overall, most of these parameters were not significantly different among the three genotypes, except for neutrophil counts (*P* = 0.05), the percent inhibition of the platelet activated by arachidonic acid (*P* = 0.05), and ADP (*P* = 0.03), and AA carriers had the lowest neutrophil counts and highest platelet inhibition rates measure by TEG via both pathways. However, when comparing drug resistance defined by dichotomized platelet aggregation inhibition rate, AA carriers had significantly higher platelet inhibition in response to ADP (*P* = 0.05) but not arachidonic acid (*P* = 0.2), arguing the association of PEAR1 minor allele (A) with suppressed platelet aggregation response in a pathway-specific manner (28). The proportions of TOAST subtypes and treatment groups were not significantly different across three genotypes (*P* = 0.61, Cochran–Mantel–Haenszel test). Three main functional outcomes measured by continuous NIHSS, mRS, and BI scale at three stages were not differed by three SNPs.

**Table 1:** Characteristics of patients by PEAR1 rs12041331 SNP.

	**AA**	**GA**	**GG**	*p*-value^*^
*n*	139	418	311	
Gender = male (%)	88 (63.3)	252 (60.3)	198 (63.7)	0.61
Age [mean (*SD*)]	69.73 (11.83)	70.72 (12.22)	71.25 (12.43)	0.47
BMI [mean (*SD*)]	26.43 (19.64)	25.47 (18.05)	24.75 (8.98)	0.64
Systolic BP [mean (*SD*)]	147.65 (20.90)	150.68 (17.94)	148.38 (18.86)	0.18
CRP [mean (*SD*)]	17.04 (36.06)	16.94 (33.08)	14.84 (30.05)	0.69
WBC [mean (*SD*)]	7.02 (1.54)	7.52 (2.33)	7.37 (1.93)	0.07
Neutrophil [mean (*SD*)]	65.51 (10.45)	67.17 (11.46)	68.58 (12.50)	0.05^**^
lymphocyte [mean (*SD*)]	27.10 (9.14)	26.15 (9.96)	25.49 (10.61)	0.327
Platelet count [mean (*SD*)]	234.89 (82.30)	235.89 (72.72)	223.32 (86.26)	0.12
Triglyceride [mean (*SD*)]	1.74 (0.95)	1.55 (1.00)	1.56 (0.94)	0.13
Cholesterol [mean (*SD*)]	4.75 (1.09)	4.67 (1.22)	4.64 (1.28)	0.68
HDL [mean (*SD*)]	1.05 (0.26)	1.05 (0.26)	1.06 (0.28)	0.99
LDL [mean (*SD*)]	3.10 (0.80)	3.02 (0.91)	3.02 (0.97)	0.67
IL6 [mean (*SD*)]	43.86 (66.86)	45.66 (82.16)	55.35 (197.83)	0.56
AA inhibition rate [mean (*SD*)]	65.25 (24.00)	62.53 (23.00)	57.79 (26.00)	0.05^**^
ADP inhibition rate [mean (*SD*)]	62.17 (27.10)	56.83 (28.50)	50.34 (29.13)	0.03^**^
Smoking history = yes (%)	62 (44.6)	160 (38.3)	120 (38.6)	0.39
Drinking history = yes (%)	22 (15.8)	56 (13.4)	44 (14.1)	0.77
Hypertension history = yes (%)	111 (79.9)	328 (78.5)	239 (76.8)	0.75
Diabetes history = yes (%)	61 (43.9)	158 (37.8)	133 (42.8)	0.27
Atrial fibrillation history = yes (%)	3 (2.2)	21 (5.0)	17 (5.5)	0.29
Coronary artery disease history = yes (%)	15 (10.8)	37 (8.9)	27 (8.7)	0.75
NIHSS_admission [mean (*SD*)]	4.32 (8.45)	4.07 (4.10)	4.44 (4.64)	0.64
BI_admission [mean (*SD*)]	84.51 (20.30)	81.90 (21.12)	81.44 (21.14)	0.34
mRS_admission [mean (*SD*)]	1.77 (1.14)	2.07 (3.07)	1.94 (1.18)	0.38
NIHSS_day 7 [mean (*SD*)]	3.25 (3.89)	3.83 (4.53)	4.14 (4.95)	0.17
BI_day 7 [mean (*SD*)]	86.57 (18.17)	83.48 (21.76)	82.65 (22.01)	0.2
mRS_day 7 [mean (*SD*)]	1.59 (1.17)	2.02 (5.00)	1.82 (1.31)	0.45
NIHSS_discharge [mean (*SD*)]	3.52 (4.92)	4.07 (5.79)	4.59 (6.57)	0.19
BI_discharge [mean (*SD*)]	85.75 (20.97)	83.01 (22.95)	81.77 (23.84)	0.24
mRS_discharge [mean (*SD*)]	1.68 (1.51)	2.17 (5.99)	1.86 (1.38)	0.41
TOAST subtype (%)				0.31
CE	3 (2.2)	18 (4.3)	12 (3.9)	
LAA	68 (48.9)	218 (52.2)	177 (56.9)	
SAO	68 (48.9)	182 (43.5)	122 (39.2)	
Aspirin resistant (%)	11 (11)	37 (12)	38 (17)	0.20
Clopidogrel resistant (%)	13 (17)	48 (21)	50 (30)	0.05^**^
Antiplatelet therapy = DAPT (%)	81 (58.3)	235 (56.2)	167 (53.7)	0.63

*SNP, single nucleotide polymorphism; BMI, body mass index; WBC, white blood cell; BP, blood pressure; CRP, C-reactive protein; HDL, high-density lipoprotein; LDL, low-density lipoprotein; IL6. Interleukin 6; AA, arachidonic acid; ADP, adenosine diphosphate; NIHSS, National Institutes of Health Stroke Scale; BI, Barthel Index; mRS, modified Rankin Scale; TOAST, the Trial of Org 10172 in Acute Stroke Treatment, LAA, large-artery atherosclerosis; SAO, small-artery occlusion; CE, cardioembolism. SD, standard deviation; DAPT, dual antiplatelet therapy.*

* *p-values were calculated using one-way analysis of variance (ANOVA) for continuous variables and Chi-Square tests for categorical variables;*

** *p-values were further corrected using false discovery rate for multiple testing*.

### Characteristics of Patients by Trial of Org 10172 in Acute Stroke Treatment and Antiplatelet Therapy

As we reported before (29) and shown in Supplementary Table 1, the increase of known inflammatory risk markers, such as counts of WBC (*P* < 0.001), neutrophil (*P* < 0.001), and lymphocyte (*P* < 0.0001), was significantly associated with the non-lacunar subtype (CE + LAA) when compared with SAO subtype. Among tested traditional risk factors, only age (*P* = 0.005) and atrial fibrillation history (*P* = 0.005) were significantly associated with the non-lacunar subtype, consistent with previous reports (29). In addition, the non-lacunar subtype was significantly associated with poor outcomes defined by all three scores regardless of stages (all *P* < 0.001). These results supported the notion that the non-lacunar subtype has a higher risk for stroke recurrence than SAO (30).

In our study, a total of 385 patients (44%) received aspirin-alone therapy, and 483 (56%) received DAPT. The proportions of antiplatelet therapy were not different among the two main TOAST subtypes (*P* = 0.84). In contrast, as shown in Supplementary Table 2, between two therapy groups, aspirin-alone had less male patients (*P* < 0.001), and the patients were significantly older (*P* < 0.001) and had lower platelet counts (*P* = 0.03), triglyceride (*P* = 0.002), and LDL (*P* = 0.04). Aspirin-alone also had lower proportions of patients who had a history of smoking (*P* < 0.001), drinking (*P* = 0.002), and atrial fibrillation (*P* = 0.04) and had more clopidogrel resistant participants (*P* < 0.001). The distribution of PEAR1 SNP and outcomes defined by dichotomized NIHSS, mRS, and BI scores at three stages were not significantly different between the two therapy groups. Interestingly, subgroup analyses showed that AA carriers treated with aspirin alone tended to have more SOA subtypes (*P* = 0.07) and more patients with favorable outcomes defined by three scoring systems (all FDR values were less than or nearly < 0.05) when compared with the DAPT group (Supplementary Table 3), suggesting a distinct role for AA genotype in response to aspirin treatment. Further examinations using the Cochran–Mantel–Haenszel test also supported that the proportions of patients, when treated with aspirin alone compared with DAPT, were significantly higher in the SAO subtype with better outcomes after controlling for PEAR1 SNP (data not shown).

### Association Between Platelet Endothelial Aggregation Receptor 1 Single-Nucleotide Polymorphisms and Stroke Outcomes

We first asked whether PEAR1 SNPs are associated with TOAST subtypes and functional outcomes measured by NIHSS, mRS, and BI at admission before antiplatelet therapy. Univariate logistic regression analyses showed that AA homozygotes were significantly associated with the SAO subtype in the patients who received aspirin-alone treatment (OR = 2.05; 95% CI, 1.11–3.83; *P* = 0.02) when compared with GG homozygotes (Table 2). No significant association was found between PEAR1 SNP and TOAST subtypes in the DAPT group (Table 2). Similarly, AA was significantly associated with favorable functional outcomes defined by all three admission scoring systems only in the aspirin-alone group but not in the DAPT group (OR = 0.46, 95% CI, 0.23–0.91, FDR = 0.05 for admission NIHSS; OR = 0.43, 95% CI, 0.23–0.81, FDR = 0.02 for admission mRS; and OR = 0.45, 95% CI, 0.24–0.4, FDR = 0.03 for admission BI, respectively; Table 2). GA was not significantly associated with either SAO or better outcomes compared with GG in all these settings (Table 2). Moreover, when we analyzed the association between SNP and outcomes within TOAST subtypes (SAO *vs*. non-lacunar), we found that AA was indeed significantly associated with better outcomes at day 7 and discharge but not at admission for all three scores only in SAO patients who had aspirin-alone treatment (Table 3). Such association was not significant in either SAO patients who had DAPT or non-lacunar patients, regardless of the type of therapies (Table 3 and Supplementary Table 4). These data suggested that in the SAO group, AA carriers are more likely sensitive to the aspirin treatment compared with GG carriers. Furthermore, we tested whether the impact of aspirin treatment on AA carriers is TOAST subtype dependent and found that aspirin alone resulted in favorable outcomes at day 7 and discharge on AA carriers in the SAO subtype but not in the non-lacunar subtype (Supplementary Table 5). In contrast, aspirin alone was not significantly associated with favorable outcomes at day 7 and discharge on GA or GG carriers, regardless of TOAST subtypes (Supplementary Table 5).

**Table 2:** Association between PEAR1 SNPs and TOAST subtype and admission outcomes in univariate logistic regression models.

		**Aspirin-alone**				**DAPT**			
**Outcome**	**PEAR1 SNPs**	**OR**	**95%**	**C.I**.	*q*-value^*^	**OR**	**95%**	**C.I**.	*P*-value^**^
TOAST	AA	2.05	1.11	3.83	0.05	1.16	0.68	1.99	0.58
SAO vs. CE + LAA	GA	1.32	0.85	2.07	0.2	1.10	0.73	1.64	0.65
NIHSS_admission	AA	0.46	0.23	0.91	0.05	0.83	0.46	1.49	0.52
Poor vs. Other	GA	0.62	0.37	1.05	0.08	1.01	0.64	1.57	0.98
mRS_admission	AA	0.43	0.23	0.81	0.02	0.90	0.52	1.59	0.72
Poor vs. Other	GA	0.87	0.54	1.39	0.6	0.87	0.57	1.32	0.53
BI_admission	AA	0.45	0.24	0.84	0.03	0.96	0.55	1.69	0.88
Poor vs. Other	GA	0.96	0.59	1.57	0.9	0.84	0.55	1.28	0.43

*SNP, single nucleotide polymorphism; TOAST, the Trial of Org 10172 in Acute Stroke Treatment; LAA, large-artery atherosclerosis; CE, cardioembolism; SAO, small-artery occlusion; OR, odds ratio; CI, confidence interval;*

* *false discovery rate (FDR) correction for multiple testing;*

** *without FDR correction for multiple testing*.

**Table 3:** Outcome association analysis between PEAR1 SNP using *univariate* logistic regression models.

		**SAO-aspirin-alone**				**Non-lacunar-aspirin-alone**			
**Outcome**		**OR**	**95%**	**C.I**.	*q*-value^*^	**OR**	**95%**	**C.I**.	*P*-value^**^
NIHSS_admission	AA vs. GG	0.54	0.22	1.32	0.40	0.61	0.18	2.42	0.45
	GA vs. GG	0.79	0.38	1.60	0.50	0.52	0.21	1.19	0.13
NIHSS_day 7	AA vs. GG	0.26	0.09	0.69	0.02	1.05	0.34	4.01	0.93
	GA vs. GG	0.93	0.46	1.86	0.80	0.64	0.30	1.33	0.20
NIHSS_discharge	AA vs. GG	0.29	0.10	0.73	0.02	1.01	0.32	3.85	0.98
	GA vs. GG	0.88	0.44	1.76	0.70	0.63	0.30	1.31	0.20
mRS_admission	AA vs. GG	0.52	0.21	1.25	0.30	0.49	0.19	1.38	0.16
	GA vs. GG	1.03	0.51	2.05	>0.9	0.85	0.41	1.74	0.60
mRS_day 7	AA vs. GG	0.23	0.07	0.65	0.02	0.72	0.28	1.96	0.50
	GA vs. GG	0.97	0.48	1.95	>0.9	0.86	0.44	1.66	0.70
mRS_discharge	AA vs. GG	0.27	0.09	0.72	0.03	0.68	0.27	1.87	0.44
	GA vs. GG	0.93	0.46	1.86	0.80	0.85	0.43	1.63	0.60
BI_admission	AA vs. GG	0.62	0.25	1.50	0.60	0.42	0.15	1.19	0.09
	GA vs. GG	1.16	0.58	2.34	0.70	0.93	0.43	2.02	0.90
BI_day 7	AA vs. GG	0.27	0.09	0.73	0.03	0.67	0.26	1.84	0.42
	GA vs. GG	0.95	0.47	1.90	0.90	0.85	0.43	1.66	0.60
BI_discharge	AA vs. GG	0.30	0.11	0.78	0.04	0.64	0.25	1.76	0.37
	GA vs. GG	0.86	0.43	1.72	0.70	0.84	0.42	1.62	0.60

*SNP, single nucleotide polymorphism; TOAST, the Trial of Org 10172 in Acute Stroke Treatment; LAA, large-artery atherosclerosis; CE, cardioembolism; SAO, small-artery occlusion; NIHSS, National Institutes of Health Stroke Scale; BI, Barthel Index; mRS, modified Rankin Scale; OR, odds ratio, CI, confidence interval;*

* *false discovery rate (FDR) correction for multiple testing;*

** *without FDR correction for multiple testing*.

Lastly, when tested in multivariate regression models, we found that AA genotype was an independent factor that was associated with better outcomes at day 7 and discharge in the SAO group who had aspirin-alone treatment after adjusted for three known risk factors that we previously reported, including age, hypertension history, and CRP (Table 4). There were no significant associations between AA homozygotes and outcomes when compared with GG in SAO patients treated with DAPT or in non-lacunar patients, regardless of treatments (Table 4 and Supplementary Table 6). Also, multivariate regression analyses showed that aspirin-alone treatment led to better outcomes at day 7 and discharge only in AA carriers with the SAO subtype after adjusting for known risk factor neutrophil–lymphocyte ratio in our study (Supplementary Table 7). Together, these results demonstrated that PEAR1 AA homozygotes had a strong association with short-term favorable functional outcomes in SAO patients treated with aspirin alone, implying for the first time a potential clinical utility of using PEAR1 rs12041331 SNP as a biomarker for targeted therapeutic strategy in patients with a subtype of acute ischemic stroke.

**Table 4:** Outcome association analysis using multivariable logistic regression models.

		**SAO-aspirin-alone**					**CE** **+** **LAA-aspirin-alone**				
**Outcomes**	**Covariates**	**OR**	**95%**	**C.I**.	***p*-value**	*q*-value^*^	**OR**	**95%**	**C.I**.	***p*-value**	*q*-value^*^
NIHSS_day 7	AA vs. GG	0.23	0.07	0.64	0.01	0.02	1.14	0.35	4.47	0.80	0.80
	GA vs. GG	0.75	0.34	1.61	0.50	0.5	0.67	0.30	1.43	0.30	0.40
	Age	1.06	1.03	1.1	<0.001	0.002	1.04	1.02	1.07	0.00	0.01
	Hypertension history	2.54	1.08	6.33	0.04	0.06	1.65	0.70	3.74	0.20	0.40
	CRP	1.37	0.99	1.92	0.06	0.07	1.25	0.93	1.70	0.15	0.40
NIHSS_discharge	AA vs. GG	0.26	0.09	0.71	0.01	0.03	1.10	0.34	4.30	0.90	0.90
	GA vs. GG	0.7	0.32	1.51	0.40	0.4	0.67	0.31	1.44	0.30	0.40
	Age	1.06	1.03	1.1	<0.001	0.001	1.04	1.02	1.07	0.00	0.01
	Hypertension history	2.53	1.08	6.26	0.04	0.06	1.67	0.71	3.77	0.20	0.40
	CRP	1.33	0.97	1.86	0.08	0.1	1.27	0.95	1.73	0.12	0.30
mRS_day 7	AA vs. GG	0.2	0.06	0.6	0.01	0.03	0.75	0.28	2.11	0.60	0.70
	GA vs. GG	0.79	0.37	1.67	0.50	0.5	0.96	0.47	1.92	0.90	0.90
	Age	1.04	1.01	1.07	0.01	0.03	1.03	1.01	1.06	0.01	0.04
	Hypertension history	3.08	1.27	8.15	0.02	0.03	2.41	1.13	5.08	0.02	0.04
	CRP	1.29	0.95	1.77	0.11	0.14	1.41	1.08	1.89	0.02	0.04
mRS_discharge	AA vs. GG	0.24	0.08	0.69	0.01	0.03	0.72	0.27	2.02	0.50	0.60
	GA vs. GG	0.75	0.35	1.58	0.40	0.4	0.95	0.47	1.91	0.90	0.90
	Age	1.04	1.01	1.08	0.01	0.03	1.03	1.01	1.06	0.01	0.03
	Hypertension history	3.07	1.28	8.11	0.02	0.03	2.40	1.14	5.04	0.02	0.03
	CRP	1.25	0.92	1.71	0.20	0.2	1.44	1.10	1.93	0.01	0.03
BI_day 7	AA vs. GG	0.25	0.08	0.72	0.01	0.03	0.70	0.26	1.98	0.50	0.60
	GA vs. GG	0.81	0.38	1.71	0.60	0.6	0.94	0.46	1.90	0.90	0.90
	Age	1.04	1.01	1.08	0.01	0.03	1.03	1.01	1.06	0.01	0.03
	Hypertension history	2.77	1.18	7	0.02	0.04	2.57	1.21	5.43	0.01	0.03
	CRP	1.13	0.83	1.54	0.40	0.5	1.36	1.04	1.82	0.03	0.05
BI_discharge	AA vs. GG	0.28	0.09	0.78	0.02	0.03	0.67	0.25	1.90	0.40	0.50
	GA vs. GG	0.69	0.32	1.48	0.30	0.3	0.93	0.46	1.88	0.80	0.80
	Age	1.05	1.02	1.09	0.00	0.01	1.03	1.01	1.06	0.01	0.03
	Hypertension history	3.34	1.38	8.86	0.01	0.03	2.56	1.21	5.39	0.01	0.03
	CRP	1.17	0.87	1.6	0.30	0.3	1.39	1.06	1.85	0.02	0.04

*LAA, large-artery atherosclerosis; CE, cardioembolism; SAO, small-artery occlusion; NIHSS, National Institutes of Health Stroke Scale; BI, Barthel Index; mRS, modified Rankin Scale; CRP, C-reactive protein; OR, odds ratio, CI, confidence interval;*

* *false discovery rate (FDR) correction for multiple testing*.

## Discussion

PEAR1 gene is a membrane protein involved in platelet activation and platelet aggregation. The results of this study showed that overall PEAR1 rs12041331 SNP did not correlate with many common basic risk factors such as patients' age, sex, level of lipid, comorbidities, and some inflammatory risk factors we previously reported except for the counts of neutrophil that was significantly lower in AA carriers. AA carriers also had a significantly higher platelet aggregation inhibition rate that measures both AA and ADP pathways, consistent with previous studies (9). PEAR1 rs12041331 SNP was not associated with TOAST subtypes or functional outcomes at admission in the entire cohort. In contrast, subgroup analyses showed that in the aspirin-alone group, the AA allele was significantly associated with SAO and functional outcomes at admission when compared with the DAPT group. Furthermore, we found that the PEAR1 AA genotype was significantly associated with favorable short-term functional outcomes only in the SAO-aspirin-alone group. To our knowledge, this was the first study to associate PEAR1 rs12041331 SNP with the TOAST subtype and functional outcomes in aspirin-treated patients with small-artery occlusion stroke.

Previously Lewis's study reported a paradoxical finding in which the rs12041331 A-allele was associated with lower post-aspirin collagen-stimulated platelet aggregation but increased cardiovascular events (11). We found that the number of rs12041331 A-allele was significantly associated with platelet aggregation inhibition in the entire cohort or subgroup analyses, regardless of the TOAST subtypes or treatment, consistent with the pieces of literature (31, 32). However, in univariable regression models, neither continual nor dichotomized AA-induced platelet aggregation inhibition was associated with functional outcomes in any of the analyses. Our findings suggest that although PEAR1 rs12041331 SNP affects platelet function and the platelet aggregation process (33, 34), the measurement platelet function or reactivity alone is not a sufficient indicator for the stork outcome risk (31, 35). The assessment of aspirin resistance through measuring platelet reactivity is highly assay dependent, and currently, no standardized or widely accepted definition of aspirin resistance exists due to the difficulty to define cutoff values for high or low on-treatment platelet reactivity (35–37). In addition, several studies showed no significant improvements in clinical outcomes with platelet function monitoring and treatments, as compared with standard antiplatelet therapy without monitoring (38, 39), further arguing the utility for tailoring treatment based on platelet response that remains to be definitively proven in clinical trials (40). In our study, the fact that rs12041331 was not associated with baseline functional outcomes but significantly associated with day 7 and discharge outcomes only in the SAO-aspirin-alone group even after adjusting for other known risk factors strongly suggests the association is a true pharmacogenetic effect and might be TOAST subtype dependent, further implying a unique undetermined role for this polymorphism in a patient group with specific TOAST subtype.

The frequency of PEAR1 AA alleles in this study was 16% (139 of 868 patients), similar to other studies reported in other ethnic populations (11). The studies on the association between PEAR1 rs12041331 and cardiovascular outcomes in the presence of aspirin treatment were very limited, and the results were controversial (41, 42). Lewis's study that reported rs12041331 A-allele carriers on aspirin appeared to have poorer outcomes than A-allele carriers that are not on aspirin in Caucasian subjects (12). However, no association was observed between rs12041331 and cardiovascular events in aspirin-treated African American or Hispanic subjects (11). To our knowledge, by far, there has been no single study focusing on the relationship of rs12041331 SNP with stroke subtypes and functional outcomes. Therefore, our study provided for the first time compelling evidence supporting genetic testing that may allow clinicians to personalize antiplatelet therapy, especially in a certain subgroup of stroke patients. The possible cell functional mechanisms that would link the PEAR1 genotype to stroke subtypes and stroke outcomes are further warranted to be investigated (43, 44).

One big limitation of our study was the small sample size in subgroup analyses such as in AA carriers with SAO subtype (*n* = 68), which led to the limited statistical power, especially in multivariable regression models. Another limitation was the lack of long-term outcome measurements, and it was difficult to compare the effect of aspirin treatment on outcome association with other studies in which either longer follow-ups or different endpoints such as death, reinfarction, stroke occurrence, or bleeding were applied (10, 11). Nonetheless, the association between this polymorphism and the functional outcome of ischemic stroke has never been studied. Therefore, our finding warrants further investigation and validation in different ethnic groups. Last, our study was only limited to one SNP of PEAR1 and other PEAR1 SNPs (45) or other gene polymorphisms (46) that could influence the patients' outcomes in combination with PEAR1 rs12041331 after aspirin treatment should be considered in the future larger study.

## Data Availability Statement

The datasets presented in this study can be found in online repositories. The names of the repository/repositories and accession number(s) can be found in the article/Supplementary Material.

## Ethics Statement

The studies involving human participants were reviewed and approved by the ethics committee of Yangpu Hospital, Tongji University School of Medicine. The patients/participants provided their written informed consent to participate in this study.

## Author Contributions

ZL, HJ, and YY carried out the studies, participated in collecting data, and drafted the manuscript. YD, DZ, and XZ performed the statistical analysis and participated in its design. JX, RM, and LH participated in the acquisition, analysis, or interpretation of data and drafted the manuscript. All authors read and approved the final manuscript.

## Conflict of Interest

The authors declare that the research was conducted in the absence of any commercial or financial relationships that could be construed as a potential conflict of interest.

## Publisher's Note

All claims expressed in this article are solely those of the authors and do not necessarily represent those of their affiliated organizations, or those of the publisher, the editors and the reviewers. Any product that may be evaluated in this article, or claim that may be made by its manufacturer, is not guaranteed or endorsed by the publisher.
