# Supplementary material for: Platelet Endothelial Aggregation Receptor 1 Polymorphism Is Associated With Functional Outcome in Small-Artery Occlusion Stroke Patients Treated With Aspirin
Source: Front Cardiovasc Med. 2021 Sep 1;8:664012. doi: 10.3389/fcvm.2021.664012 (PMC8440843; doi:10.3389/fcvm.2021.664012)
Supplement: Supplementary file 1 [file Table_1.docx]

| **Supplemental Table 1 Characteristics of patients by TOAST subtypes** | | |  |
| --- | --- | --- | --- |
|  | CE+LAA | SAO | P value* |
| n | 496 | 372 |  |
| Gender = male (%) | 315 (63.5) | 223 (59.9) | 0.32 |
| Age (mean (SD)) | 71.91 (12.58) | 69.20 (11.59) | 0.005** |
| BMI (mean (SD)) | 24.63 (7.47) | 26.26 (21.84) | 0.18 |
| Systolic BP (mean (SD)) | 149.60 (18.44) | 149.11 (19.21) | 0.72 |
| CRP (mean (SD)) | 18.41 (35.94) | 13.02 (26.50) | 0.06** |
| WBC (mean (SD)) | 7.68 (2.25) | 6.98 (1.76) | <0.001** |
| Neutrophil (mean (SD)) | 69.15 (12.09) | 64.95 (10.76) | <0.001** |
| lymphocyte (mean (SD)) | 24.53 (10.22) | 28.24 (9.47) | <0.001** |
| Platelet Count (mean (SD)) | 235.24 (86.25) | 225.34 (68.42) | 0.09 |
| Triglyceride (mean (SD)) | 1.59 (1.08) | 1.57 (0.80) | 0.70 |
| Cholesterol (mean (SD)) | 4.71 (1.27) | 4.62 (1.15) | 0.26 |
| HDL (mean (SD)) | 1.06 (0.26) | 1.05 (0.28) | 0.94 |
| LDL (mean (SD)) | 3.06 (0.95) | 3.00 (0.86) | 0.35 |
| IL6 (mean (SD)) | 52.84 (167.35) | 43.52 (67.78) | 0.31 |
| AA inhibition rate (mean (SD)) | 60.80 (24.45) | 61.82 (24.32) | 0.60 |
| ADP inhibition rate (mean (SD)) | 56.24 (27.65) | 54.18 (30.28) | 0.44 |
| Smoking history = yes (%) | 200 (40.3) | 142 (38.2) | 0.57 |
| Drinking history = yes (%) | 76 (15.3) | 46 (12.4) | 0.25 |
| Hypertension history = yes (%) | 393 (79.2) | 285 (76.6) | 0.40 |
| Diabetes history = yes (%) | 192 (38.7) | 160 (43.0) | 0.23 |
| Atrial Fibrillation history = yes (%) | 33 (6.7) | 8 (2.2) | 0.005** |
| Coronary artery disease history = yes (%) | 46 (9.3) | 33 (8.9) | 0.93 |
| NIHSS_admission = poor (%) | 419 (84.5) | 217 (58.3) | <0.001** |
| mRS_admission = poor (%) | 380 (76.8) | 189 (50.8) | <0.001** |
| NIHSS_day7 = poor (%) | 396 (81.0) | 181 (49.5) | <0.001** |
| mRS_day7 = poor (%) | 358 (73.2) | 155 (42.3) | <0.001** |
| NIHSS_discharge = poor (%) | 401 (80.8) | 184 (49.5) | <0.001** |
| mRS_discharge= poor (%) | 364 (73.4) | 158 (42.5) | <0.001** |
| BI_admission = poor (%) | 386 (78.0) | 201 (54.0) | <0.001** |
| BI_day7 = poor (%) | 360 (73.6) | 159 (43.4) | <0.001** |
| BI_discharge = poor (%) | 366 (73.8) | 160 (43.0) | <0.001** |
| Aspirin resistant (%) | 47 (13) | 39 (14) | 0.95 |
| Clopidogrel resistant (%) | 60 (22) | 51 (26) | 0.29 |
| Antiplatelet therapy = DAPT (%) | 278 (56.0) | 205 (55.1) | 0.84 |
| PEAR1 rs12041331 (%) |  |  | 0.15 |
| AA | 71 (14.3) | 68 (18.3) |  |
| GA | 236 (47.6) | 182 (48.9) |  |
| GG | 189 (38.1) | 122 (32.8) |  |

TOAST, the Trial of Org 10172 in Acute Stroke Treatment, LAA, large-artery atherosclerosis; SAO, small-artery occlusion; CE, cardioembolism.; BMI, body mass index; BP, blood pressure; WBC, white blood cell; CRP, C-reactive protein; HDL, high-density lipoprotein; LDL, low-density lipoprotein; IL6. Interleukin 6; AA, arachidonic acid; ADP, adenosine diphosphate ; NIHSS, National Institutes of Health Stroke Scale; BI, Barthel Index; mRS, modified Rankin Scale;. SD, standard deviation; DAPT, dual antiplatelet therapy. *p values were calculated using one-way analysis of variance (ANOVA) for continuous variables and Chi-Square tests for categorical variables; ** p values were further corrected using false discovery rate for multiple testing
