# Supplementary material for: Platelet Endothelial Aggregation Receptor 1 Polymorphism Is Associated With Functional Outcome in Small-Artery Occlusion Stroke Patients Treated With Aspirin
Source: Front Cardiovasc Med. 2021 Sep 1;8:664012. doi: 10.3389/fcvm.2021.664012 (PMC8440843; doi:10.3389/fcvm.2021.664012)
Supplement: Supplementary file 2 [file Table_2.docx]

| Supplemental Table 2 Characteristics of patients by antiplatelet therapy | | |  |
| --- | --- | --- | --- |
|  | Aspirin | DAPT | P value* |
| n | 385 | 483 |  |
| Gender = male (%) | 198 (51.4) | 340 (70.4) | <0.001** |
| Age (mean (SD)) | 74.97 (12.24) | 67.39 (11.14) | <0.001** |
| BMI (mean (SD)) | 25.24 (11.20) | 25.49 (18.58) | 0.84 |
| Systolic BP (mean (SD)) | 148.58 (19.11) | 150.01 (18.49) | 0.30 |
| CRP (mean (SD)) | 14.90 (29.92) | 17.28 (34.51) | 0.31 |
| WBC (mean (SD)) | 7.29 (2.26) | 7.47 (1.94) | 0.23 |
| Neutrophil (mean (SD)) | 67.73 (11.65) | 67.16 (11.81) | 0.50 |
| lymphocyte (mean (SD)) | 25.41 (10.00) | 26.60 (10.14) | 0.10 |
| Platelet Count (mean (SD)) | 225.04 (84.64) | 236.31 (74.65) | 0.03** |
| Triglyceride (mean (SD)) | 1.45 (0.78) | 1.69 (1.09) | 0.002** |
| Cholesterol (mean (SD)) | 4.59 (1.30) | 4.73 (1.15) | 0.09 |
| HDL (mean (SD)) | 1.07 (0.27) | 1.05 (0.26) | 0.33 |
| LDL (mean (SD)) | 2.97 (0.97) | 3.09 (0.86) | 0.04** |
| IL6 (mean (SD)) | 55.85 (184.42) | 43.26 (71.90) | 0.17 |
| AA inhibition rate (mean (SD)) | 60.71 (24.09) | 61.70 (24.64) | 0.61 |
| ADP inhibition rate (mean (SD)) | 45.78 (28.63) | 61.23 (27.26) | <0.001** |
| Smoking history = yes (%) | 113 (29.4) | 229 (47.4) | <0.001** |
| Drinking history = yes (%) | 36 (9.4) | 86 (17.8) | 0.002** |
| Hypertension history = yes (%) | 302 (78.4) | 376 (77.8) | 0.90 |
| Diabetes history = yes (%) | 145 (37.7) | 207 (42.9) | 0.14 |
| Atrial Fibrillation history = yes (%) | 26 (6.8) | 15 (3.1) | 0.04** |
| Coronary artery disease history = yes (%) | 32 (8.3) | 47 (9.7) | 0.55 |
| NIHSS_admission = poor (%) | 286 (74.3) | 350 (72.5) | 0.60 |
| mRS_admission = poor (%) | 257 (66.9) | 312 (64.6) | 0.52 |
| NIHSS_day7 = poor (%) | 249 (66.2) | 328 (68.5) | 0.53 |
| mRS_day7 = poor (%) | 223 (59.3) | 290 (60.5) | 0.77 |
| NIHSS_discharge = poor (%) | 255 (66.2) | 330 (68.3) | 0.56 |
| mRS_discharge= poor (%) | 229 (59.5) | 293 (60.7) | 0.78 |
| BI_admission = poor (%) | 270 (70.3) | 317 (65.6) | 0.16 |
| BI_day7 = poor (%) | 228 (60.6) | 291 (60.8) | 1.00 |
| BI_discharge = poor (%) | 233 (60.5) | 293 (60.7) | 1.00 |
| TOAST subtype (%) |  |  | 0.64 |
| CE | 17 (4.4) | 16 (3.3) |  |
| LAA | 201 (52.2) | 262 (54.2) |  |
| SAO | 167 (43.4) | 205 (42.4) |  |
| Aspirin resistant (%) | 33 (11) | 53 (15) | 0.22 |
| Clopidogrel resistant (%) | 65 (36) | 46 (16) | <0.001** |
| PEAR1 rs12041331 (%) |  |  | 0.63 |
| AA | 58 (15.1) | 81 (16.8) |  |
| GA | 183 (47.5) | 235 (48.7) |  |
| GG | 144 (37.4) | 167 (34.6) |  |

DAPT, dual antiplatelet therapy ; BMI, body mass index; BP, blood pressure; WBC, white blood cell; CRP, C-reactive protein; HDL, high-density lipoprotein; LDL, low-density lipoprotein; IL6. Interleukin 6; AA, arachidonic acid; ADP, adenosine diphosphate ; NIHSS, National Institutes of Health Stroke Scale; BI, Barthel Index; mRS, modified Rankin Scale;. SD, standard deviation; TOAST, the Trial of Org 10172 in Acute Stroke Treatment, LAA, large-artery atherosclerosis; SAO, small-artery occlusion; CE, cardioembolism. *p values were calculated using one-way analysis of variance (ANOVA) for continuous variables and Chi-Square tests for categorical variables; ** p values were further corrected using false discovery rate for multiple testing
