# Supplementary material for: Platelet Endothelial Aggregation Receptor 1 Polymorphism Is Associated With Functional Outcome in Small-Artery Occlusion Stroke Patients Treated With Aspirin
Source: Front Cardiovasc Med. 2021 Sep 1;8:664012. doi: 10.3389/fcvm.2021.664012 (PMC8440843; doi:10.3389/fcvm.2021.664012)
Supplement: Supplementary file 3 [file Table_3.docx]

Supplemental Table 3 Characteristics of patient TOAST subtype and outcomes by PEAR1 SNP and antiplatelet therapy

|  |  | Aspirin alone | |  |  |  | DAPT |  |  |
| --- | --- | --- | --- | --- | --- | --- | --- | --- | --- |
|  | AA | GA | GG | q-value* |  | AA | GA | GG | p-value** |
| n | 58 | 183 | 144 |  |  | 81 | 235 | 167 |  |
| TOAST subtype = SAO (%) | 32 (55.2) | 81 (44.3) | 54 (37.5) | 0.07 |  | 36 (44.4) | 101 (43.0) | 68 (40.7) | 0.83 |
| NIHSS_admission = poor (%) | 38 (65.5) | 132 (72.1) | 116 (80.6) | 0.06 |  | 56 (69.1) | 172 (73.2) | 122 (73.1) | 0.76 |
| NIHSS_day 7 = poor (%) | 29 (51.8) | 118 (65.9) | 102 (72.3) | 0.03 |  | 56 (69.1) | 162 (69.5) | 110 (66.7) | 0.82 |
| NIHSS_discharge = poor (%) | 30 (51.7) | 120 (65.6) | 105 (72.9) | 0.03 |  | 56 (69.1) | 163 (69.4) | 111 (66.5) | 0.82 |
| mRS_admission = poor (%) | 30 (51.7) | 125 (68.3) | 102 (71.3) | 0.03 |  | 52 (64.2) | 149 (63.4) | 111 (66.5) | 0.82 |
| mRS_day 7 = poor (%) | 23 (41.1) | 109 (60.9) | 91 (64.5) | 0.03 |  | 53 (65.4) | 136 (58.4) | 101 (61.2) | 0.52 |
| mRS_discharge = poor (%) | 24 (41.4) | 111 (60.7) | 94 (65.3) | 0.03 |  | 53 (65.4) | 138 (58.7) | 102 (61.1) | 0.56 |
| BI_admission = poor (%) | 32 (55.2) | 133 (72.7) | 105 (73.4) | 0.03 |  | 54 (66.7) | 150 (63.8) | 113 (67.7) | 0.71 |
| BI_day 7 = poor (%) | 24 (42.9) | 111 (62.0) | 93 (66.0) | 0.03 |  | 54 (66.7) | 135 (57.9) | 102 (61.8) | 0.36 |
| BI_discharge = poor (%) | 25 (43.1) | 112 (61.2) | 96 (66.7) | 0.03 |  | 53 (65.4) | 137 (58.3) | 103 (61.7) | 0.50 |

TOAST, the Trial of Org 10172 in Acute Stroke Treatment, LAA, large-artery atherosclerosis; SAO, small-artery occlusion; NIHSS, National Institutes of Health Stroke Scale; BI, Barthel Index; mRS, modified Rankin Scale; DAPT, dual antiplatelet therapy. * False discovery rate (FDR) correction for multiple testing; ** Pearson’s Chi-squared test.
