# Supplementary material for: Platelet Endothelial Aggregation Receptor 1 Polymorphism Is Associated With Functional Outcome in Small-Artery Occlusion Stroke Patients Treated With Aspirin
Source: Front Cardiovasc Med. 2021 Sep 1;8:664012. doi: 10.3389/fcvm.2021.664012 (PMC8440843; doi:10.3389/fcvm.2021.664012)
Supplement: Supplementary file 4 [file Table_4.docx]

Supplemental Table 4 Outcome association analysis between PEAR1 AA and GG genotype using univariable logistic regression models

|  |  | SAO-DAPT |  |  | Non.lacunar-DAPT | | | |
| --- | --- | --- | --- | --- | --- | --- | --- | --- |
| Outcome | OR | 95% | C.I. | P value* | OR | 95% | C.I. | P value* |
| NIHSS_admission | 0.78 | 0.35 | 1.77 | 0.55 | 0.96 | 0.39 | 2.53 | 0.93 |
| NIHSS_day 7 | 1.30 | 0.58 | 2.94 | 0.53 | 1.11 | 0.46 | 2.91 | 0.82 |
| NIHSS_discharge | 1.26 | 0.56 | 2.84 | 0.58 | 1.17 | 0.49 | 3.05 | 0.73 |
| mRS_admission | 1.11 | 0.49 | 2.52 | 0.80 | 0.79 | 0.36 | 1.77 | 0.55 |
| mRS_day 7 | 1.64 | 0.73 | 3.74 | 0.24 | 0.99 | 0.45 | 2.26 | 0.99 |
| mRS_discharge | 1.58 | 0.70 | 3.61 | 0.27 | 1.03 | 0.47 | 2.34 | 0.94 |
| BI_admission | 1.32 | 0.58 | 3.04 | 0.51 | 0.74 | 0.34 | 1.68 | 0.47 |
| BI_day 7 | 1.83 | 0.81 | 4.22 | 0.15 | 0.94 | 0.43 | 2.15 | 0.88 |
| BI_discharge | 1.58 | 0.70 | 3.61 | 0.27 | 0.98 | 0.45 | 2.23 | 0.96 |

SAO, small-artery occlusion; DAPT, dual antiplatelet therapy.; NIHSS, National Institutes of Health Stroke Scale; BI, Barthel Index; mRS, modified Rankin Scale ;OR = Odds Ratio, CI = Confidence Interval; * without FDR correction for multiple testing.
