# Supplementary material for: Platelet Endothelial Aggregation Receptor 1 Polymorphism Is Associated With Functional Outcome in Small-Artery Occlusion Stroke Patients Treated With Aspirin
Source: Front Cardiovasc Med. 2021 Sep 1;8:664012. doi: 10.3389/fcvm.2021.664012 (PMC8440843; doi:10.3389/fcvm.2021.664012)
Supplement: Supplementary file 5 [file Table_5.docx]

Supplemental Table 5 Outcome association analysis between Aspirin alone and DAPT using univariable logistic regression models among PEAR1 SNPs

|  |  |  | SAO sbutype | | | | Non.lacunar | | | |
| --- | --- | --- | --- | --- | --- | --- | --- | --- | --- | --- |
| SNP | Treatment | outcome | Ratio | 95% | C.I. | P.Value* | Ratio | 95% | C.I. | P.Value* |
| AA | Aspirin alone vs DAPT | NIHSS_admission | 0.89 | 0.34 | 2.33 | 0.82 | 1.19 | 0.33 | 4.88 | 0.80 |
|  |  | NIHSS_day 7 | 0.27 | 0.09 | 0.77 | **0.02** | 1.19 | 0.33 | 4.88 | 0.80 |
|  |  | NIHSS_discharge | 0.30 | 0.10 | 0.82 | **0.02** | 1.19 | 0.33 | 4.88 | 0.80 |
|  |  |  |  |  |  |  |  |  |  |  |
|  |  | mRS_admission | 0.48 | 0.18 | 1.26 | 0.14 | 0.91 | 0.32 | 2.69 | 0.87 |
|  |  | mRS_day 7 | 0.16 | 0.05 | 0.48 | **0.002** | 0.82 | 0.28 | 2.43 | 0.71 |
|  |  | mRS_discharge | 0.18 | 0.06 | 0.53 | **0.003** | 0.82 | 0.28 | 2.43 | 0.71 |
|  |  |  |  |  |  |  |  |  |  |  |
|  |  | BI_admission | 0.49 | 0.18 | 1.29 | 0.15 | 0.91 | 0.32 | 2.69 | 0.87 |
|  |  | BI_day 7 | 0.18 | 0.05 | 0.52 | **0.002** | 0.82 | 0.28 | 2.43 | 0.71 |
|  |  | BI_discharge | 0.22 | 0.07 | 0.63 | **0.01** | 0.82 | 0.28 | 2.43 | 0.71 |
|  |  |  |  |  |  |  |  |  |  |  |
| GA | Aspirin alone vs DAPT | NIHSS_admission | 1.04 | 0.57 | 1.88 | 0.91 | 0.87 | 0.44 | 1.74 | 0.69 |
|  |  | NIHSS_day 7 | 0.92 | 0.51 | 1.66 | 0.78 | 0.78 | 0.41 | 1.47 | 0.43 |
|  |  | NIHSS_discharge | 0.86 | 0.48 | 1.54 | 0.61 | 0.83 | 0.44 | 1.56 | 0.56 |
|  |  |  |  |  |  |  |  |  |  |  |
|  |  | mRS_admission | 1.31 | 0.73 | 2.37 | 0.36 | 1.26 | 0.68 | 2.37 | 0.46 |
|  |  | mRS_day 7 | 1.26 | 0.69 | 2.29 | 0.45 | 1.04 | 0.58 | 1.88 | 0.89 |
|  |  | mRS_discharge | 1.17 | 0.65 | 2.12 | 0.60 | 1.06 | 0.59 | 1.90 | 0.85 |
|  |  |  |  |  |  |  |  |  |  |  |
|  |  | BI_admission | 1.48 | 0.82 | 2.69 | 0.19 | 1.70 | 0.90 | 3.32 | 0.11 |
|  |  | BI_day 7 | 1.32 | 0.73 | 2.40 | 0.36 | 1.14 | 0.64 | 2.06 | 0.66 |
|  |  | BI_discharge | 1.17 | 0.65 | 2.12 | 0.60 | 1.16 | 0.65 | 2.09 | 0.62 |
|  |  |  |  |  |  |  |  |  |  |  |
| GG | Aspirin alone vs DAPT | NIHSS_admission | 1.29 | 0.62 | 2.72 | 0.50 | 1.87 | 0.80 | 4.61 | 0.16 |
|  |  | NIHSS_day 7 | 1.35 | 0.66 | 2.78 | 0.42 | 1.25 | 0.59 | 2.72 | 0.56 |
|  |  | NIHSS_discharge | 1.31 | 0.64 | 2.68 | 0.47 | 1.37 | 0.65 | 2.96 | 0.41 |
|  |  |  |  |  |  |  |  |  |  |  |
|  |  | mRS_admission | 1.03 | 0.50 | 2.12 | 0.93 | 1.46 | 0.72 | 3.01 | 0.30 |
|  |  | mRS_day 7 | 1.13 | 0.55 | 2.33 | 0.74 | 1.13 | 0.58 | 2.22 | 0.71 |
|  |  | mRS_discharge | 1.09 | 0.53 | 2.24 | 0.81 | 1.23 | 0.64 | 2.40 | 0.53 |
|  |  |  |  |  |  |  |  |  |  |  |
|  |  | BI_admission | 1.05 | 0.51 | 2.16 | 0.90 | 1.62 | 0.78 | 3.45 | 0.20 |
|  |  | BI_day 7 | 1.22 | 0.59 | 2.51 | 0.59 | 1.15 | 0.59 | 2.27 | 0.69 |
|  |  | BI_discharge | 1.18 | 0.57 | 2.42 | 0.66 | 1.25 | 0.64 | 2.45 | 0.52 |

SAO, small-artery occlusion; DAPT, dual antiplatelet therapy.; NIHSS, National Institutes of Health Stroke Scale; BI, Barthel Index; mRS, modified Rankin Scale; OR = Odds Ratio, CI = Confidence Interval; * without FDR correction for multiple testing.
