# Supplementary material for: Platelet Endothelial Aggregation Receptor 1 Polymorphism Is Associated With Functional Outcome in Small-Artery Occlusion Stroke Patients Treated With Aspirin
Source: Front Cardiovasc Med. 2021 Sep 1;8:664012. doi: 10.3389/fcvm.2021.664012 (PMC8440843; doi:10.3389/fcvm.2021.664012)
Supplement: Supplementary file 6 [file Table_6.docx]

Supplemental Table 6 Outcome association analysis using multivariable logistic regression models

|  |  |  | SAO-DAPT |  |  |  | CE+LAA-DAPT |  |  |
| --- | --- | --- | --- | --- | --- | --- | --- | --- | --- |
| Outcomes | Covariates | OR | 95% C.I. | p value | q-value** | OR | 95% C.I. | p value | q-value* |
| NIHSS_day 7 | AA vs GG | 1.31 | 0.58, 3.02 | 0.50 | 0.80 | 1.11 | 0.46, 2.92 | 0.80 | >0.9 |
|  | GA vs GG | 1.38 | 0.74, 2.59 | 0.30 | 0.80 | 1.00 | 0.51, 1.94 | >0.9 | >0.9 |
|  | Age | 1.00 | 0.98, 1.03 | 0.80 | 0.80 | 1.02 | 0.99, 1.05 | 0.20 | >0.9 |
|  | Hypertension History (Yes) | 1.41 | 0.74, 2.71 | 0.30 | 0.80 | 1.07 | 0.49, 2.19 | 0.90 | >0.9 |
|  | CRP | 1.04 | 0.81, 1.35 | 0.80 | 0.80 | 1.09 | 0.85, 1.42 | 0.50 | >0.9 |
|  |  |  |  |  |  |  |  |  |  |
| NIHSS_discharge | AA vs GG | 1.28 | 0.56, 2.94 | 0.60 | 0.80 | 1.18 | 0.49, 3.09 | 0.70 | >0.9 |
|  | GA vs GG | 1.35 | 0.73, 2.53 | 0.30 | 0.80 | 1.01 | 0.52, 1.95 | >0.9 | >0.9 |
|  | Age | 1.00 | 0.97, 1.03 | >0.9 | >0.9 | 1.02 | 0.99, 1.04 | 0.30 | >0.9 |
|  | Hypertension History (Yes) | 1.44 | 0.75, 2.77 | 0.30 | 0.80 | 0.98 | 0.45, 2.01 | >0.9 | >0.9 |
|  | CRP | 1.06 | 0.82, 1.37 | 0.70 | 0.80 | 1.10 | 0.85, 1.43 | 0.50 | >0.9 |
|  |  |  |  |  |  |  |  |  |  |
| mRS_day 7 | AA vs GG | 1.80 | 0.78, 4.19 | 0.20 | 0.60 | 1.01 | 0.46, 2.33 | >0.9 | >0.9 |
|  | GA vs GG | 0.92 | 0.49, 1.74 | 0.80 | 0.80 | 0.88 | 0.48, 1.59 | 0.70 | 0.90 |
|  | Age | 1.01 | 0.98, 1.04 | 0.50 | 0.80 | 1.01 | 0.99, 1.04 | 0.20 | 0.60 |
|  | Hypertension History (Yes) | 0.87 | 0.45, 1.68 | 0.70 | 0.80 | 0.86 | 0.43, 1.67 | 0.70 | 0.90 |
|  | CRP | 1.18 | 0.91, 1.54 | 0.20 | 0.60 | 1.19 | 0.95, 1.51 | 0.14 | 0.60 |
|  |  |  |  |  |  |  |  |  |  |
| mRS_discharge | AA vs GG | 1.75 | 0.77, 4.06 | 0.20 | 0.50 | 1.06 | 0.48, 2.42 | 0.90 | 0.90 |
|  | GA vs GG | 0.90 | 0.48, 1.69 | 0.70 | 0.70 | 0.93 | 0.51, 1.67 | 0.80 | 0.90 |
|  | Age | 1.01 | 0.98, 1.03 | 0.70 | 0.70 | 1.01 | 0.99, 1.04 | 0.30 | 0.80 |
|  | Hypertension History (Yes) | 0.89 | 0.47, 1.73 | 0.70 | 0.70 | 0.83 | 0.41, 1.59 | 0.60 | 0.90 |
|  | CRP | 1.20 | 0.93, 1.57 | 0.20 | 0.50 | 1.19 | 0.95, 1.51 | 0.14 | 0.70 |
|  |  |  |  |  |  |  |  |  |  |
| BI_day 7 | AA vs GG | 2.05 | 0.89, 4.83 | 0.10 | 0.50 | 0.96 | 0.43, 2.20 | >0.9 | >0.9 |
|  | GA vs GG | 0.93 | 0.49, 1.76 | 0.80 | 0.80 | 0.81 | 0.44, 1.47 | 0.50 | 0.80 |
|  | Age | 1.01 | 0.99, 1.04 | 0.40 | 0.60 | 1.01 | 0.99, 1.04 | 0.20 | 0.60 |
|  | Hypertension History (Yes) | 0.80 | 0.41, 1.55 | 0.50 | 0.60 | 0.86 | 0.43, 1.67 | 0.70 | 0.80 |
|  | CRP | 1.20 | 0.92, 1.57 | 0.20 | 0.50 | 1.16 | 0.92, 1.46 | 0.20 | 0.60 |
|  |  |  |  |  |  |  |  |  |  |
| BI_discharge | AA vs GG | 1.75 | 0.77, 4.06 | 0.20 | 0.50 | 1.00 | 0.45, 2.29 | >0.9 | >0.9 |
|  | GA vs GG | 0.90 | 0.48, 1.69 | 0.70 | 0.70 | 0.85 | 0.47, 1.54 | 0.60 | 0.80 |
|  | Age | 1.01 | 0.98, 1.03 | 0.70 | 0.70 | 1.01 | 0.99, 1.04 | 0.30 | 0.80 |
|  | Hypertension History (Yes) | 0.89 | 0.47, 1.73 | 0.70 | 0.70 | 0.83 | 0.41, 1.59 | 0.60 | 0.80 |
|  | CRP | 1.20 | 0.93, 1.57 | 0.20 | 0.50 | 1.16 | 0.92, 1.46 | 0.20 | 0.80 |

LAA, large-artery atherosclerosis; CE, cardioembolism ; SAO, small-artery occlusion; NIHSS, National Institutes of Health Stroke Scale; BI, Barthel Index; mRS, modified Rankin Scale; OR,Odds Ratio, CI , Confidence Interval; * False discovery rate (FDR) correction for multiple testing
