# Supplementary material for: Platelet Endothelial Aggregation Receptor 1 Polymorphism Is Associated With Functional Outcome in Small-Artery Occlusion Stroke Patients Treated With Aspirin
Source: Front Cardiovasc Med. 2021 Sep 1;8:664012. doi: 10.3389/fcvm.2021.664012 (PMC8440843; doi:10.3389/fcvm.2021.664012)
Supplement: Supplementary file 7 [file Table_7.docx]

| **Supplemental Table 7 Outcome association with aspirin alone treatment vs DAPT analysis using multivariable logistic regression models in patients with PEAR1 AA genotype** | | | | | | | | |
| --- | --- | --- | --- | --- | --- | --- | --- | --- |
|  |  |  |  |  |  |  |  |  |
|  |  |  | SAO |  |  |  | CE+LAA |  |
| Outcomes | Covariates | OR | 95% C.I. | p value* | q-value** | OR | 95% C.I. | p value* |
| NIHSS_admission | Aspirin vs DAPT | 1.02 | 0.36, 2.86 | >0.9 | >0.9 | 1.10 | 0.25, 5.32 | >0.9 |
|  | N.L.Ratio | 1.21 | 0.94, 1.80 | 0.20 | 0.40 | 3.68 | 1.33, 15.1 | 0.04 |
|  |  |  |  |  |  |  |  |  |
| NIHSS_day 7 | Aspirin vs DAPT | 0.22 | 0.06, 0.74 | 0.02 | 0.04 | 1.04 | 0.25, 4.81 | >0.9 |
|  | N.L.Ratio | 1.65 | 1.12, 3.18 | 0.07 | 0.07 | 2.39 | 1.12, 7.89 | 0.10 |
|  |  |  |  |  |  |  |  |  |
| NIHSS_discharge | Aspirin vs DAPT | 0.26 | 0.08, 0.83 | 0.03 | 0.06 | 1.04 | 0.25, 4.81 | >0.9 |
|  | N.L.Ratio | 1.55 | 1.09, 2.84 | 0.08 | 0.08 | 2.39 | 1.12, 7.89 | 0.10 |
|  |  |  |  |  |  |  |  |  |
| mRS_admission | Aspirin vs DAPT | 0.50 | 0.18, 1.40 | 0.20 | 0.40 | 1.05 | 0.33, 3.45 | >0.9 |
|  | N.L.Ratio | 1.05 | 0.82, 1.35 | 0.70 | 0.70 | 1.36 | 1.03, 2.21 | 0.10 |
|  |  |  |  |  |  |  |  |  |
| mRS_day 7 | Aspirin vs DAPT | 0.13 | 0.03, 0.45 | 0.00 | 0.01 | 0.91 | 0.29, 3.00 | 0.90 |
|  | N.L.Ratio | 1.22 | 0.94, 1.69 | 0.20 | 0.20 | 1.26 | 0.99, 1.90 | 0.14 |
|  |  |  |  |  |  |  |  |  |
| mRS_discharge | Aspirin vs DAPT | 0.16 | 0.04, 0.52 | 0.00 | 0.01 | 0.91 | 0.29, 3.00 | 0.90 |
|  | N.L.Ratio | 1.19 | 0.92, 1.62 | 0.20 | 0.20 | 1.26 | 0.99, 1.90 | 0.14 |
|  |  |  |  |  |  |  |  |  |
| BI_admission | Aspirin vs DAPT | 0.51 | 0.18, 1.43 | 0.20 | 0.40 | 1.05 | 0.33, 3.45 | >0.9 |
|  | N.L.Ratio | 1.06 | 0.84, 1.41 | 0.60 | 0.60 | 1.36 | 1.03, 2.21 | 0.10 |
|  |  |  |  |  |  |  |  |  |
| BI_day 7 | Aspirin vs DAPT | 0.15 | 0.04, 0.50 | 0.00 | 0.01 | 0.91 | 0.29, 3.00 | 0.90 |
|  | N.L.Ratio | 1.19 | 0.92, 1.66 | 0.20 | 0.20 | 1.26 | 0.99, 1.90 | 0.14 |
|  |  |  |  |  |  |  |  |  |
| BI_discharge | Aspirin vs DAPT | 0.21 | 0.06, 0.64 | 0.01 | 0.02 | 0.91 | 0.29, 3.00 | 0.90 |
|  | N.L.Ratio | 1.16 | 0.91, 1.58 | 0.20 | 0.20 | 1.26 | 0.99, 1.90 | 0.14 |

LAA, large-artery atherosclerosis; CE, cardioembolism ; SAO, small-artery occlusion; NIHSS, National Institutes of Health Stroke Scale; BI, Barthel Index; mRS, modified Rankin Scale; OR,Odds Ratio, CI , Confidence Interval; * without FDR correction; ** False discovery rate (FDR) correction for multiple testing
